# Estrogen Leads to Reversible Hair Cycle Retardation through Inducing Premature Catagen and Maintaining Telogen

**DOI:** 10.1371/journal.pone.0040124

**Published:** 2012-07-05

**Authors:** Hui-min Hu, Shou-bing Zhang, Xiao-hua Lei, Zhi-li Deng, Wei-xiang Guo, Zhi-fang Qiu, Shuang Liu, Xin-yue Wang, He Zhang, En-kui Duan

**Affiliations:** 1 State Key Laboratory of Reproductive Biology, Institute of Zoology, Chinese Academy of Sciences, Beijing, China; 2 Graduate University of Chinese Academy of Sciences, Beijing, China; 3 West North Agri-forestry University, Yangling, Shaanxi, China; Clermont Université, France

## Abstract

Estrogen dysregulation causes hair disorder. Clinical observations have demonstrated that estrogen raises the telogen/anagen ratio and inhibits hair shaft elongation of female scalp hair follicles. In spite of these clinical insights, the properties of estrogen on hair follicles are poorly dissected. In the present study, we show that estrogen induced apoptosis of precortex cells and caused premature catagen by up-regulation of TGF β2. Immediately after the premature catagen, the expression of anagen chalone BMP4 increased. The up-regulation of BMP4 may further function to prevent anagen transition and maintain telogen. Interestingly, the hair follicle stem cell niche was not destructed during these drastic structural changes caused by estrogen. Additionally, dermal papilla cells, the estrogen target cells in hair follicles, kept their signature gene expressions as well as their hair inductive potential after estrogen treatment. Retention of the characteristics of both hair follicle stem cells and dermal papilla cells determined the reversibility of the hair cycle suppression. These results indicated that estrogen causes reversible hair cycle retardation by inducing premature catagen and maintaining telogen.

## Introduction

Most areas of the mammalian skin, which functions as a protective barrier against physical injuries and pathogens, are covered by hairs. Each hair follicle (HF) is a complex mini-organ consisting of specialized cells of both epithelial and dermal origins [1]. Distinct from other organs, HFs undergo continuous growth and regression cycles [2]. The hair cycle is artificially divided into three different stages–growth stage (anagen), regression stage (catagen) and resting stage (telogen), depending on HF morphology and cell behavior [3], [4]. During hair cycle, the distal one third of the HF (the permanent part) maintains its structure almost unchanged. However, the proximal two thirds of the HF (the cycling part) is destructed and rebuilt during each cycle [5].

Two important cell populations in HF, hair follicle stem cells (HFSCs) and dermal papilla (DP) cells, are required for initiating the first step of a new hair cycle [6], [7]. The outer root sheath (ORS) portion of the bulge that locates between sebaceous gland and arrector pili muscle is known to be the HFSC pool [8], [9]. At the onset of anagen, DP activates HFSCs to grow downwards along ORS to form hair matrix [10]. Subsequently, the abundant transit-amplifying cells in hair matrix migrate inward to the center of the hair column and undergo terminally differentiation to form hair shaft [11]. Precortex cells below the proximal hair club is the transitional form between matrix cells and terminally differentiated hair shaft cells [12]. They are also the major cell type undergoing apoptosis during full and late catagen (catagen V, VI and VII) [13], [14]. DP also contributes to anagen-catagen transition by reducing proliferation and inducing apoptosis [15], [16].

BMP (bone morphogenetic protein) signaling plays a suppressive role in telogen-anagen transition [17]. BMP4, a member of this family, is down-regulated in DP and secondary hair germ during telogen-anagen transition. The essential role of BMP inhibitor Noggin in anagen induction also supports the role of BMP signaling in retaining telogen [18]. It has been confirmed that the postnatal telogen skin contains an endogenous inhibitor of telogen-anagen transition, the so-called chalone. The effects of BMP4 on postnatal telogen HF verify the identity of BMP4 as the chalone [18].

It has long been recognized that hair cycle is associated with the activity cycle of the gonads [19]. The effects of androgen on androgenetic alopecia have been well established. Androgen shortens duration of the anagen stage of scalp hair follicle. The normal anagen lasts from 2 to as long as 7 years. Whereas, in men with androgenetic alopecia, the duration of anagen will decrease from years to months or even weeks [20]. As the result, the hair shafts are much thinner than normal. Then the terminal hairs were replaced by vellus hairs. Contrast to the shortened anagen, the duration of telogen remained the same or lengthens. The ratio of anagen/telogen hairs decreases (from a normal 6 to 8∶1 ratio to an abnormal 0.1 to 3∶1 ratio) [21]. The hairs of telogen stage are more easily to be plucked when brushing or shampooing. The consequences of androgen dysregulation are much thinner hairs and much more telogen hairs, and these hairs highly tend to shed. The progressive hair cycle changes caused androgenetic alopecia finally.

Besides androgen, the effects of estrogen on hair disorder has been well established as well. Due to high levels of plasma estrogens, pregnant women display a slower rate of replacement of spontaneous hair loss or plucked hair [22]. Their scalp hairs demonstrated an increased telogen/anagen ratio [23]. The hair shaft elongation of organ cultured female scalp HFs is also inhibited by estrogen [24], [25]. Taken the regionality of human scalp into account, much more studies employed murine model to investigate the effects of estrogen on HFs and uncovered some of the mechanisms. In 1996, Smart et al. proposed that estrogen suppresses murine hair growth and the inhibitor of estrogen has an entirely opposite effect [26]. Ohlsson et al. further demonstrated that estrogen receptor (ER) α, but not ER β, is involved in the regulation of the HF cycle [27]. Since the molecular mechanism that respectively manipulates each stage of the hair cycle is quite different from each other, whether the estrogen caused alopecia is a process of telogen-maintaining or anagen-telogen transition is a key question before further mechanism exploration. In the present study, we report that estrogen treatment caused premature onset of catagen, characterized by apoptosis of precortex cells through up-regulation of TGF β2. Subsequently, expression of the anagen chalone, BMP4 increased and the estrogen induced telogen was maintained. Although the long-lasting estrogen treatment caused alopecia, the HFSCs were not ablated. Furthermore, the DP cells showed no involution of their signature gene expressions after estrogen treatment. This suggested that the potential of DP cells to induce hair regrowth was reserved. For this reason, after estrogen treatment repealed, the hairs could regrow. Based on these results, the estrogen induced hair cycle retardation is reversible.

## Results

### Estrogen Induces Premature Catagen and then Maintains Telogen

**Figure 1:**
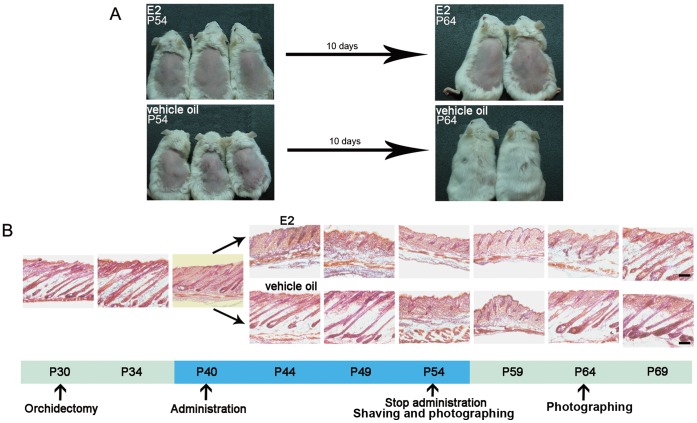
Estrogen induced premature catagen and suppressed HFs at telogen in a reversible manner. (A) Estrogen administration inhibited hair regrowth. At the tenth day after hair shaving (P64), the estrogen treated mice remained bald, whereas the backs of the vehicle oil treated mice were fully covered by hairs. (B) Estrogen induced premature catagen and arrested HFs at telogen. After orchidectomy, HFs entered a long-lasting anagen (P30–49). In contrast, just after four days of 17β-estradiol injection (P44), the hairs were obliged to enter catagen from anagen, and stayed at telogen until at least ten days after stopping injection (P54–64). However, 15 days after withdrawal of the 17β-estradiol treatment (P69), the mice had their hairs regrown. The period of 17β-estradiol or vehicle oil treatment is highlighted by blue. E2: 17β-estradiol; HF: hair follicle**.** Scale bar: 100 µm.

To elucidate the effect of 17β-estradiol on hair cycle, male CD1 mice were orchidectomized at postnatal day 30 (P30). After 10 days of recovery, the mice were randomly divided into two groups. One group was injected with 0.66 µg 17β-estradiol/mouse/day for the next 15 days (P40–54). As control, the other group was injected with the same volume of sesame oil. Dorsal hairs of the two groups were shaved at P54, and the appearance of hair regrowth was photographed at the tenth day after hair shaving (P64, Figure 1A). The treatment process is summarized in Figure 1B, as the period of estrogen administration is marked in blue. The estrogen-treated mice remained bald, whereas the backs of the vehicle oil-treated mice were fully covered by hairs.

To describe the effect of 17β-estradiol on hair cycle in detail, we investigated the hair cycles of differently treated mice utilizing time-lapse histology analysis (Figure 1B). As previous reports, immediately after orchiectomy, hairs of the mice entered a long-term anagen [27] (P30–49). However, the 17β-estradiol treated group entered catagen from anagen after four days of administration (P44), and stayed in telogen for at least ten days after stopping injection (P54–64). Interestingly, 15 days after withdrawal of 17β-estradiol administration (P69), the estrogen treated mice had their hairs regrown.

**Figure 2:**
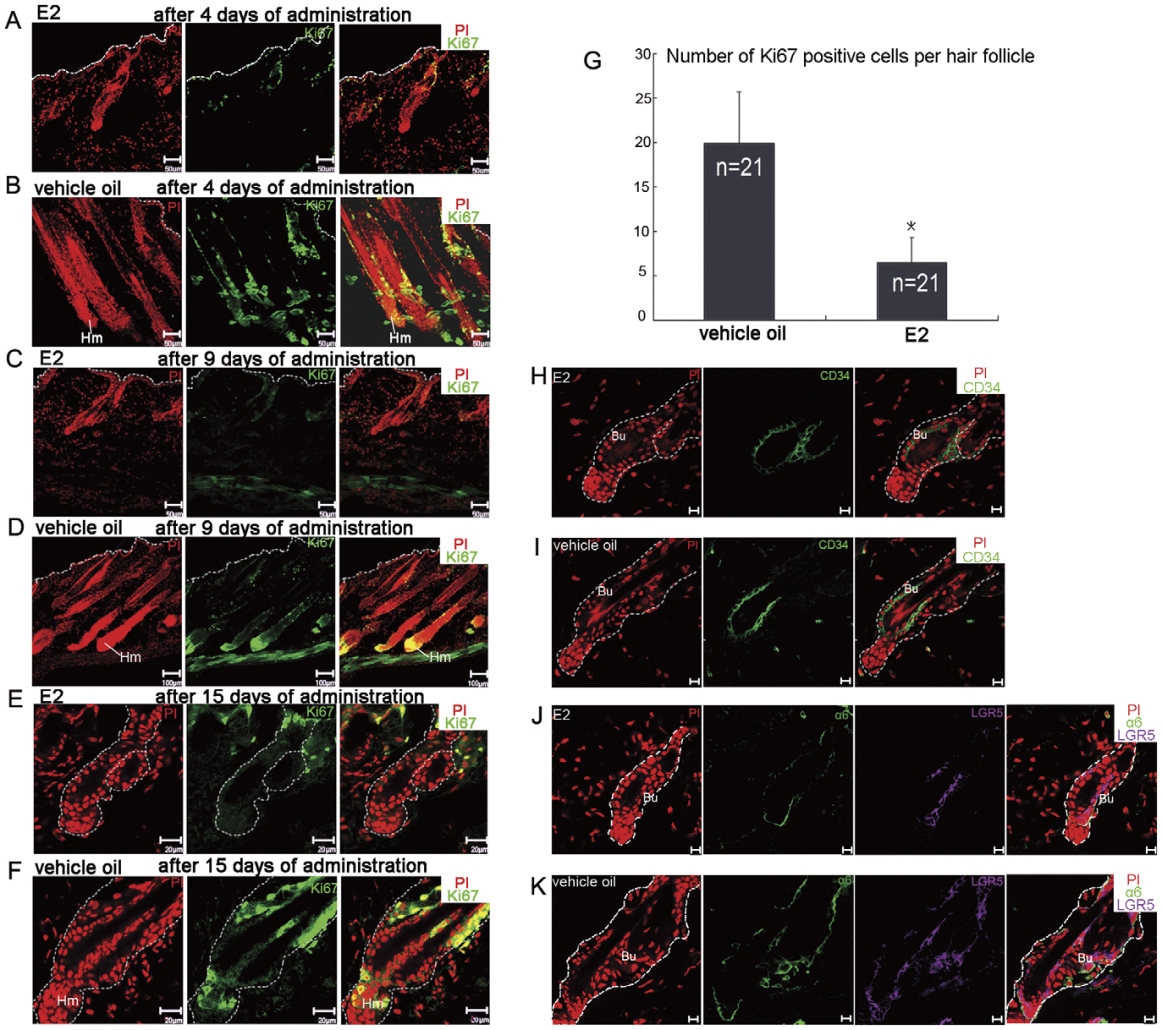
Estrogen inhibited proliferation of hair matrix cells, while HFSCs were reserved. HFs of the estrogen treated mice with a typical catagen phenotype (A) and telogen phenotype (C) contained just several Ki67 positive cells after 4 (A) and 9 (C) days of treatment. HFs of the vehicle oil treated mice manifested typical anagen phenotype with lengthening hair fibers and large bulb after 4 (B) and 9 (D) days of treatment, and abundant Ki67 positive cells were detected in hair matrix. (E) After another 6 days of continuous treatment, few cells were activated in HFs of the estrogen treated mice. (F) HFs of the vehicle oil treated mice had gone through a whole hair cycle at the fifteenth day of treatment. They stayed at telogen, yet still contained a mass of Ki67 positive cells in their distal ORS. (G) The histogram gives a quantitative view over the different count of proliferating cells in differently treated HFs at P54. 21 HFs of each treatment were investigated (*, P<0.05, independent sample T test). (H) At the fifteenth day of treatment, the HFSC marker CD34 expressed in the bulge of estrogen treated mice in a similar manner as vehicle oil treated mice (I). Integrin α6 and LGR5 were detected both in HFs of the estrogen treated mice (J) and the vehicle oil treated mice (K). Hm: hair matrix; Bu: bulge. Dotted line indicates epithelium (A–D) or the boundary of HF (E–F, H–K). Propidium iodide-labeled nuclei are in red. E2: 17β-estradiol; HFSCs: hair follicle stem cell; HF: hair follicle; ORS: outer root sheath; LGR5: Leucine-rich G protein-coupled receptor 5. Scale bars: 50 µm (A–C); 100 µm (D); 20 µm (E–F); 10 µm (H–K).

To detect the cell proliferation dynamics during the drastic HF morphology changes, we did immunofluorescence staining using proliferative marker Ki67 antibody at three different time points (after 4, 9 and 15 days of treatment). Immunofluorescence results revealed that the HFs of estrogen treated mice which displayed a typical catagen (after 4 days of treatment, Figure 2A) or telogen phenotype (after 9 days of treatment, Figure 2C), contained just several Ki67 positive cells. In contrast, HFs of the control mice manifested a representative anagen phenotype with lengthening hair fibers and swelling hair bulbs after 4 or 9 days of treatment, and abundant Ki67 positive cells were detected in hair matrix (Figure 2B and 2D). After another 6 days of continuous administration, few cells were activated in HFs of the estrogen treated mice which manifested a representative telogen phenotype (Figure 2E). In contrast, HFs of the control group had gone through a whole HF cycle and remained at telogen. Nevertheless, they were still with a mass of Ki67 positive cells in their distal ORS (Figure 2F). The count of Ki67 positive cells per HF in the differently treated groups at the day stopping administration (P54) gave us a direct view (Figure 2G).

### Upon Estrogen Treatment, HFSCs and DP Retain their Characteristics

The noted suppressive effect of estrogen on hair growth called our attention to the fate of HFSCs, since the prognoses of alopecias with or without destruction of HFSCs are quite different. Were the HFSCs still reserved in their niche, they could be reactivated to trigger trichogenesis [28]. So we assayed the expression of the putative HFSC markers. Several proteins have been reported as HFSC markers. Glycoprotein CD34 is reported expressing in mouse keratinocytes of the HF bulge coinciding with the label-retaining cells [29]. A member of the integrin family, integrin α6 highly expresses in basal layer, including HFSCs niche–the bulge [30]. Besides these two commonly used markers, LGR5 (Leucine-rich G protein-coupled receptor 5) was recently reported marking a population of cells which are multipotent and proliferating actively [31]. In the present work, after 15 days of estrogen administration, CD34 and LGR5 were detected on the bulge cell membrane of the estrogen treated mice HFs (Figure 2H and 2J) in a similar manner as the control mice (Figure 2I and 2K). Integrin α6 was detected expressing in HF basal layers of both the control mice (Figure 2K) and the estrogen treated mice (Figure 2J).

**Figure 3:**
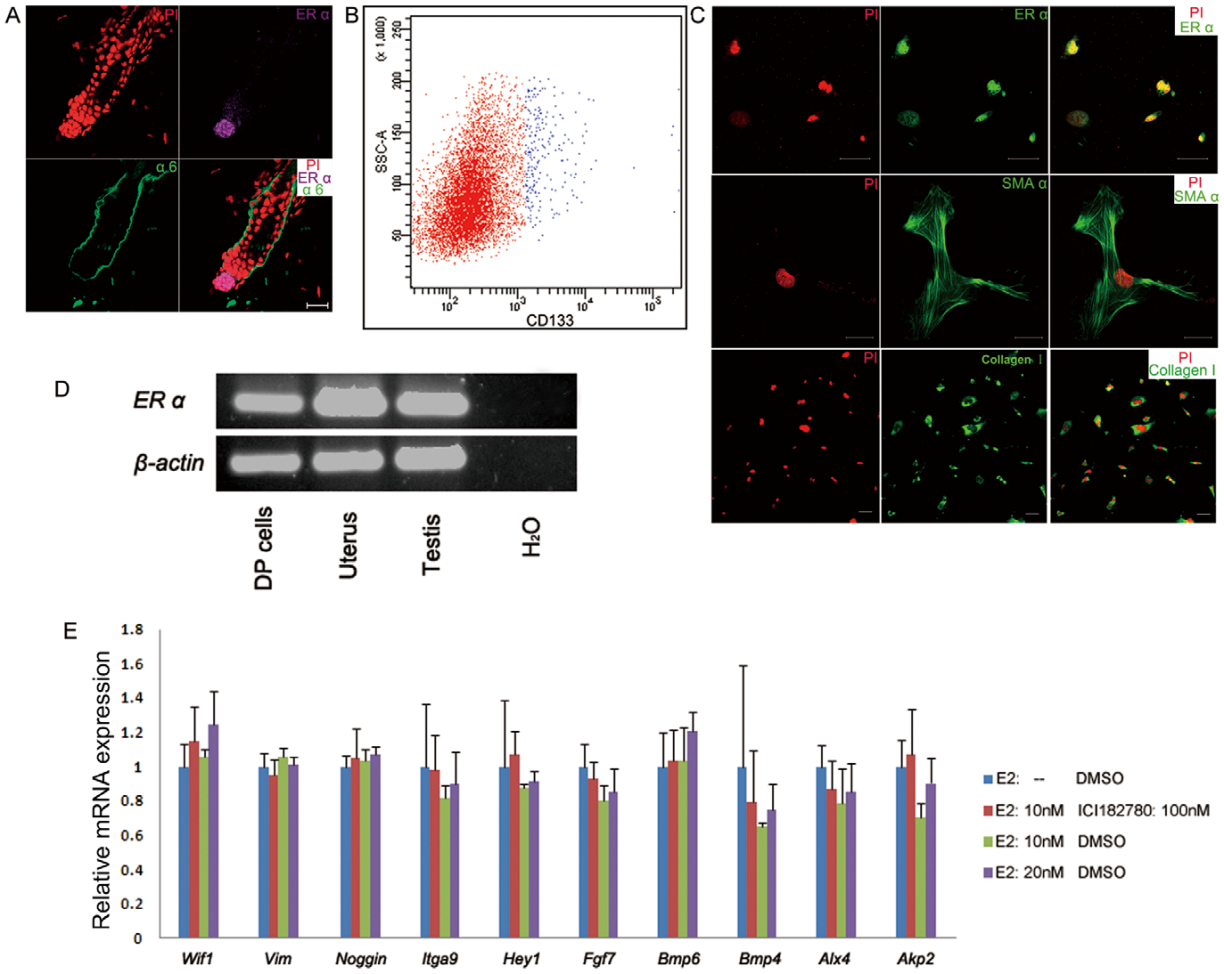
The hair inductive potential of DP cells was not compromised upon estrogen. (A) ER α located at DP of adult HF. (B) DP cells were isolated from neonatal mice dermis following published protocol. CD133 positive cells were collected for estrogen treatment and further assay. (C) Sorted DP cells expressed ER α. As a mesenchymal cell population, they also expressed SMA α and collagen I. (D) Sorted DP cells expressed ER α on mRNA level. Mice uterus and testis were employed as positive control while H_2_O as negative control. (E) Real-time PCR was conducted on a battery of DP signature genes. After exposed respectively to 10 nM, 20 nM of 17β-estradiol or 10 nM of 17β-estradiol and 100 nM ICI 182780, the expression levels of these DP signature genes were not significantly altered (P<0.01 was considered to be significant, one-way ANOVA analysis). Error bars indicate SD of triplicate independent experiments. E2: 17β-estradiol; DP: dermal papilla; ER: estrogen receptor; HF: hair follicle; SMA α: smooth muscle actin α. Scale bars: 20 µm (A); 50 µm (C).

As ER α, but not ER β had been demonstrated involved in estrogen-induced hair cycle suppression, we examined the location of ER α in mice HF. The double-staining of antibodies against ER α and basal layer marker integrin α6 showed that DP cells were ER α positive (Figure 3A). In the light of their importance on conducting hair cycle, DP cells had been comprehensively profiled on molecular level. The DP signature genes were evidenced vital for the retaining of cultured DP cells’ potential to induce hair growth after transplantation [32], [33]. In order to explore the effects of estrogen on DP cells’ inductive potential, DP cells were isolated and exposed to estrogen to detect the expression spectrum of their signature genes. DP cells were sorted from neonatal mice according to reported protocol [34] (Figure 3B). Immunocytochemistry and RT-PCR results revealed that the isolated cells expressed ER α (Figure 3C and 3D) as well as smooth muscle actin α (SMA α) [35] and Collagen I [36], [37], which were both reported exclusively expressed in the dermis of skin and commonly used as characteristic markers of DP cells (Figure 3C). Considering that cultured DP cells would lose HF inductive capacity after several passages [34], cells of passage 1 were used in our study. They were exposed to 10 nM, 20 nM of 17β-estradiol or 10 nM of 17β-estradiol combined with 100 nM of ICI 182780 for 24 hours to test the effects of estrogen on their HF inductive potential (Figure 3E). Real-time PCR was employed to detect the expressions of well established DP signature genes that function to maintain DP characteristic. No significant (P<0.01) change was found in the expression of ten signature genes. The result is based on three independent experiments.

### During Estrogen Induced Premature Onset of Catagen, TGF β2 is Up-regulated and Induces Apoptosis of Hair Shaft Precursor Cells

**Figure 4:**
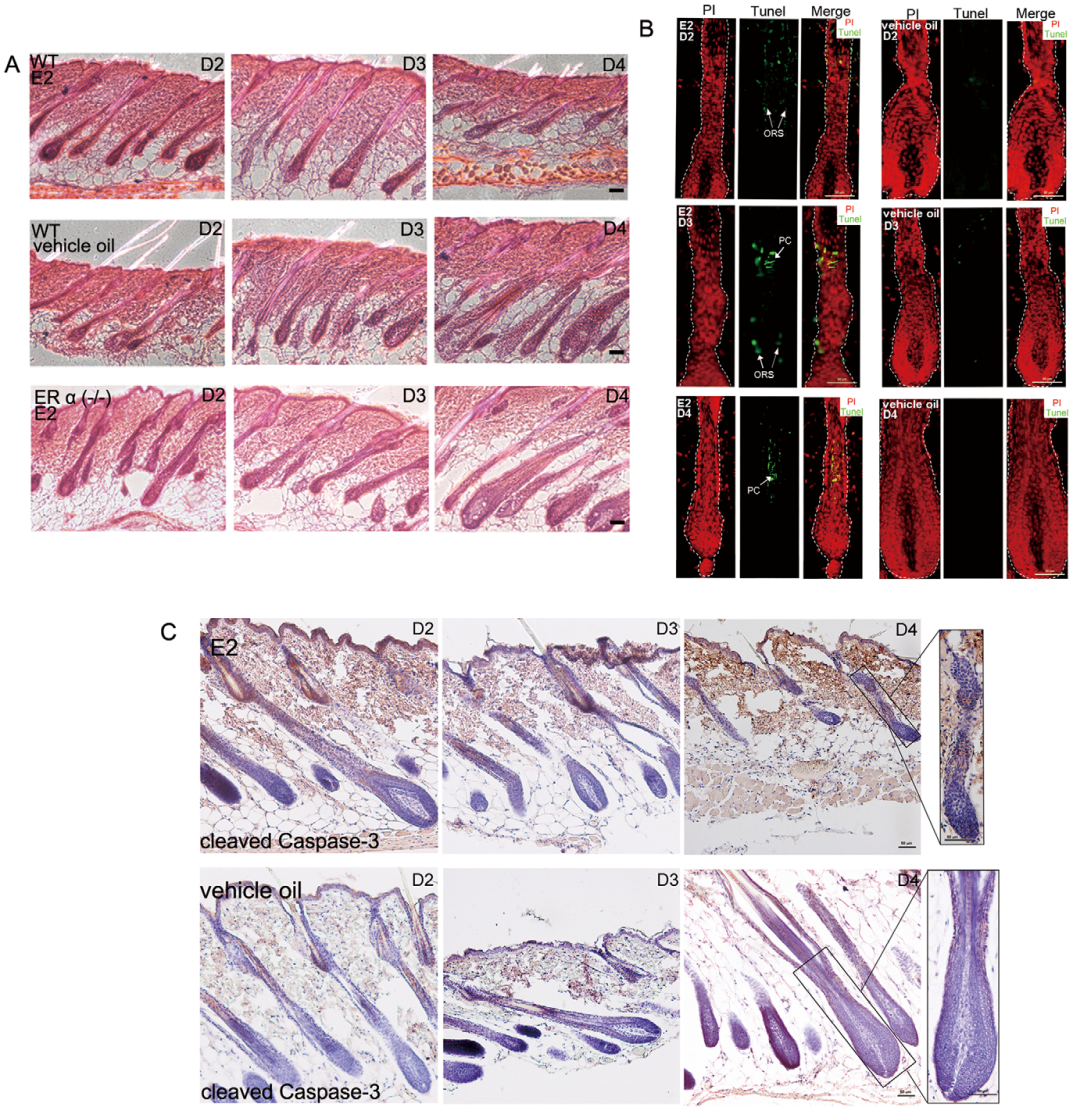
The premature catagen induced by estrogen was accompanied with advance apoptosis of precortex cells. (A) Premature catagen happened to the WT mice HFs as soon as the 4th day of estrogen treatment (P44). Yet estrogen treatment showed no effect on HFs of ER α (−/−) ones. (B) After 3 days or 4 days of estrogen treatment, precortex cells of the HFs showed Tunel-positive. The days of treatment are indicated. (C) Compared with HFs of the vehicle oil treated mice, the HFs upon estrogen showed cleaved caspase-3 expression in precortex region. Boxed areas are magnified in right-hand panels. Propidium iodide-labeled nuclei are in red. PC: precortex; ORS: outer root sheath; E2: 17β-estradiol; HF: hair follicle; ER: estrogen receptor. Scale bar: 50 µm.

The previous reports about estrogen induced hair cycle suppression were all based on long period estrogen administration model [26], [27]. In order to explore the mechanism underlying the estrogen induced hair cycle arrest, we tried to confine the exact time point that estrogen starts to perform its suppressive effect. We orchidectomized wild type (WT) and estrogen receptor alpha null (ER α −/−) mice, and treated them with estrogen or vehicle oil and obtained time-lapse sections after 2, 3 and 4 days of administration (the time of orchidectomy and recovery was as same as the model in Figure 1B). As our observation, after 4 days of estrogen administration, HFs of WT mice showed typical catagen V–VII morphology, while HFs of the vehicle oil treated WT mice and estrogen treated ER α (−/−) mice stayed at stages of anagen IV–VI (Figure 4A).

In order to illustrate the cell behaviors in the estrogen-induced regressive HFs, we performed an apoptosis assay. After 3 and 4 days of estrogen administration, precortex cells showed a significant Tunel (terminal deoxynucleotide transferase dUTP fluorescein nick end-labeling) staining (Figure 4B). Additionally, the apoptosis marker cleaved caspase-3 was also detected in the precortex region of HFs upon 4 days of estrogen administration (Figure 4C).

**Figure 5:**
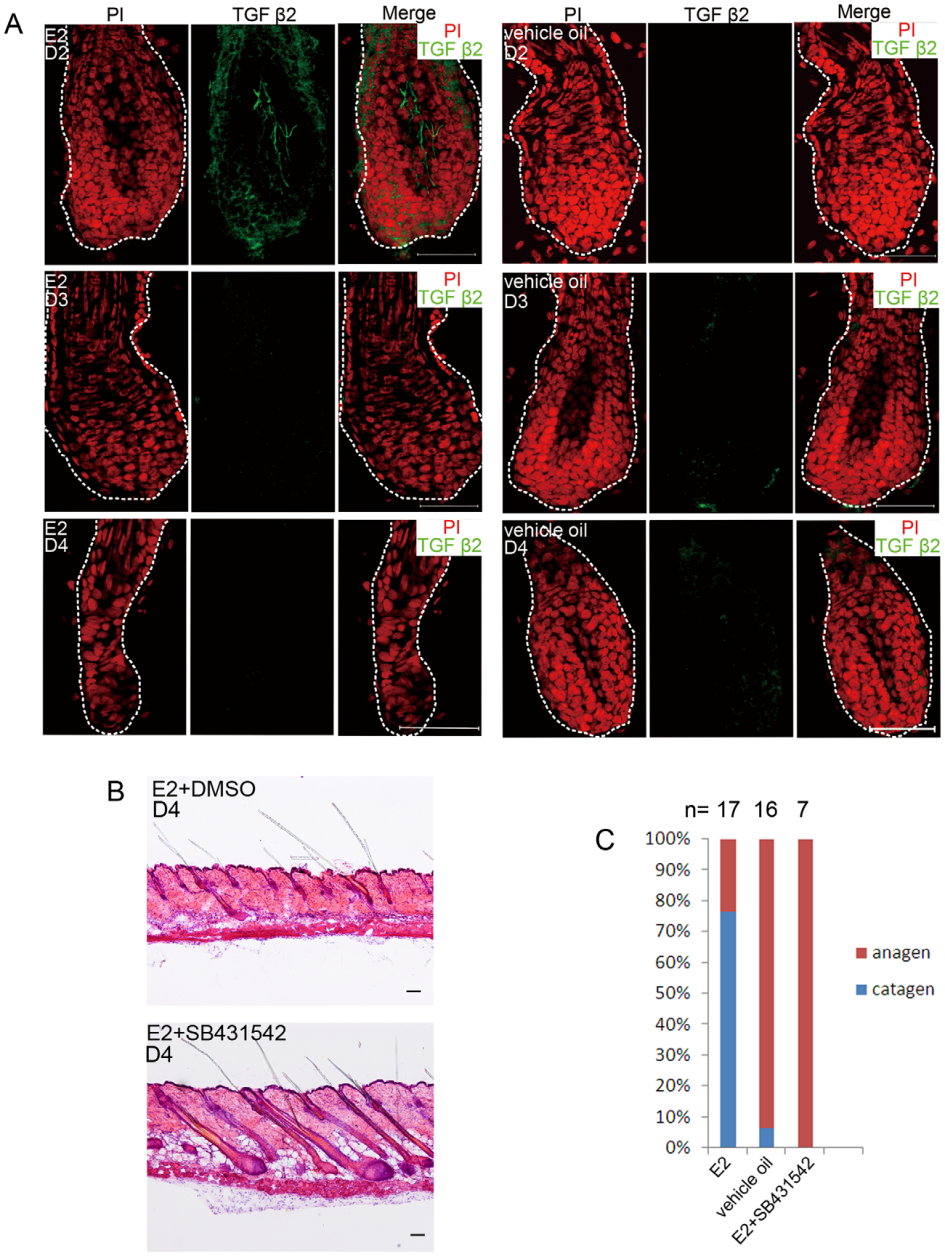
TGF β2 acted as a downstream effector in the estrogen induced hair cycle arrest. (A) TGF β2 expressed in HFs of the estrogen treated mice. The skin was derived after 2, 3 and 4 days of estrogen or vehicle oil administration. Note TGF β2 up-regulation in DP of the estrogen treated mice HF after 2 days of administration. The days of treatment are indicated. Propidium iodide-labeled nuclei are in red. (B) The pretreatment with a specific TGF β signalling inhibitor SB431542 rescued the hair cycle arrest caused by estrogen. The estrogen+DMSO mice were with catagen HFs, whereas the estrogen+SB431542 ones with anagen HFs. (C) Statistics of phenotypes of differently treated mice. Numbers above the bars indicate number of mice used for each assay. E2: 17β-estradiol; HF: hair follicle; DP: dermal papilla. Scale bar: 50 µm (A), 100 µm (B).

Molecular mechanisms of apoptosis control during catagen have been dissected and functions of several signalings have been demonstrated in this process [14]. Among these signals, only TGF β2 expresses in DP [33]. Considering the location of ER α at DP, we supposed that TGF β2 might be the downstream effector in estrogen induced hair cycle arrest. Corresponding to the early signaling role that TGF β2 plays in triggering catagen [16], we found a remarkable expression of TGF β2 in DP as soon as the second day of estrogen administration, although the hairs were still with typical anagen morphology (Figure 5A). By contrast, the vehicle oil treated group showed negligible TGF β2 expression in edge of the hair bulb and no expression in DP.

To confirm that TGF β2 is exactly a downstream effector in estrogen induced hair cycle arrest, the specific inhibitor of TGF β signaling, SB431542, was employed to rescue the suppressive effect of estrogen. SB431542 (10 mg/kg) or vehicle (DMSO) was i.p. injected 1 h before estrogen administration. After 4 days of treatment, HFs of the SB431542+estrogen mice were in anagen, whereas the DMSO+estrogen ones were in catagen (Figure 5B). The statistics is showed in Figure 5C.

### BMP4 Expression Increases in the HFs of Estrogen Treated Mice, and Telogen is Sustained

**Figure 6:**
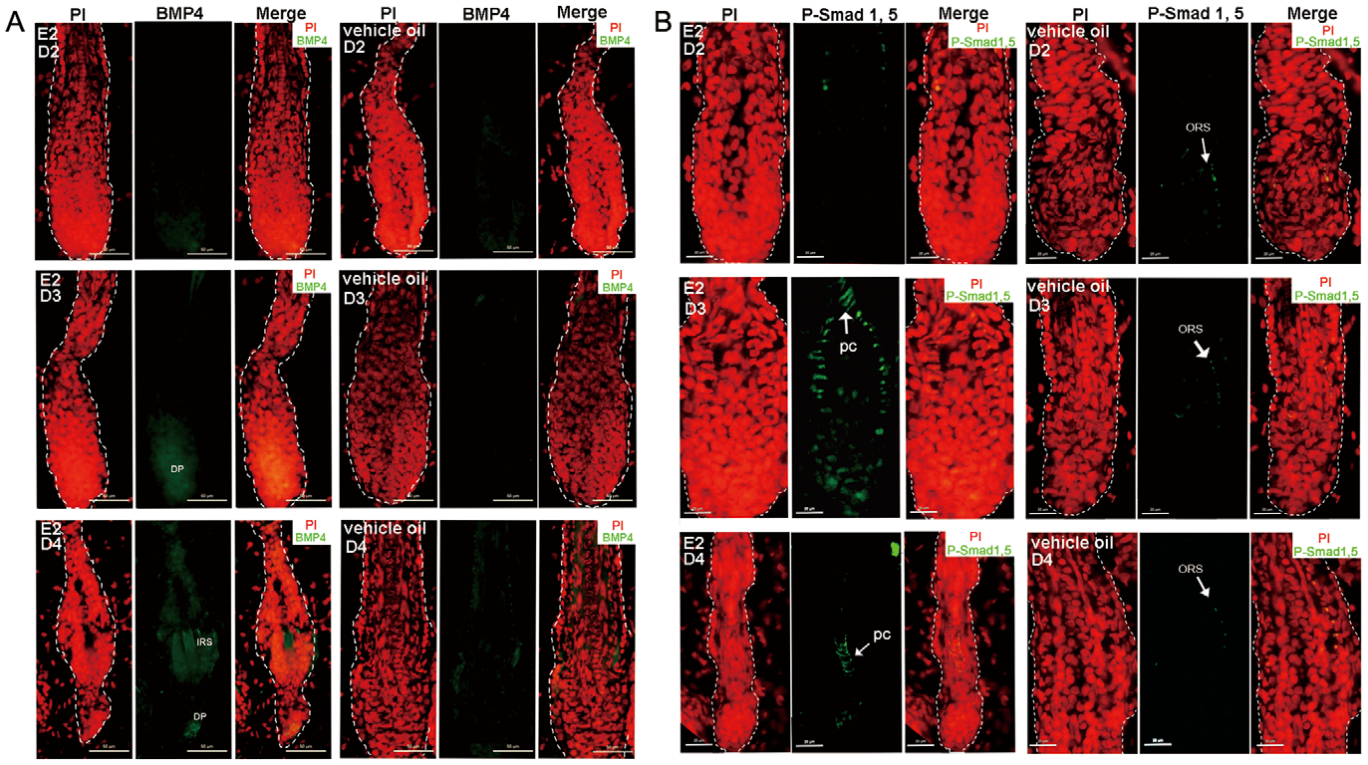
HFs of the estrogen treated mice showed activation of BMP pathway. (A) Increase expression of BMP4 in hair keratinocytes and DP cells after 3 and 4 days of estrogen treatment vs. HFs of the oil treatment mice. (B) After 3 and 4 days of estrogen treatment, a high phosphorylation level of BMP signaling effector, Smad 1/5, was detected in precortex, while the control ones had negligible P-Smad 1/5 expression in ORS. The days of treatment are indicated. Propidium iodide-labeled nuclei are in red. ORS: outer root sheath; IRS: inner root sheath; E2: 17β-estradiol; HF: hair follicle; DP: dermal papilla**.** Scale bar: 50 µm (A); 20 µm (B).

In the process of estrogen induced hair cycle arrest, the premature onset of catagen was followed by a lasting telogen. We assayed expression of BMP4, which functions in sustaining telogen and blocking anagen transition [17]. As shown in Figure 6A, after 3 and 4 days of estrogen administration, BMP4 expressed in DP mesenchymal cells. The outcome of BMP signaling activation is cytoplasmic phosphorylation of Smad 1/5/8, enabling their interaction with Smad 4 and translocation to the nucleus, where their target genes are then transcribed [17]. P-Smad 1/5 was slightly detected in ORS of the vehicle oil treated mice (Figure 6B). Compared with the control ones, the estrogen treated group showed distinct P-Smad 1/5 staining in precortex regions after 3 and 4 days of treatment. Lower magnification figures of BMP4 and P-Smad 1/5 expressions after 4 days of treatment are in supplementary (Figure S1).

## Discussion

**Figure 7:**
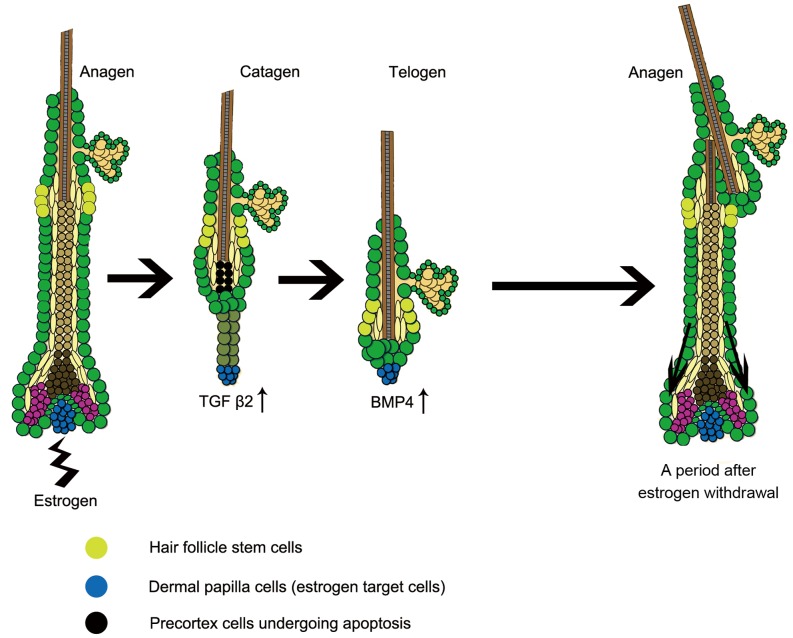
Model of the mechanism of estrogen induces hair cycle arrest. The intrinsic or extrinsic estrogen activates ER α on DP cells. Then the expression of TGF β2 is up-regulated and the precortex cells undergo apoptosis, which triggers premature onset of catagen. Immediately after the catagen, the anagen chalone BMP4 increases its expression in DP and hair matrix. As the result, telogen is sustained. Although estrogen plays a suppressive role on hair growth, the HFSCs are kept from damage. After estrogen treatment withdrawal, the reserved HFSCs were reactivated and the hairs could regenerate. ER: estrogen receptor; DP: dermal papilla; HFSC: hair follicle stem cell.

Estrogen has been demonstrated playing important role in hair growth and skin physiology [26]. However, the cell behaviors and the potential signaling pathways involved in estrogen induced hair cycle suppression have been seldom studied. Since cell behavior and signaling are strictly dependent on the stage of hair cycle, it is a pivotal question to confirm the alteration of hair cycle induced by estrogen treatment. Previous works have proved the estrogen effect on hair cycle, based on long period estrogen administration. However, weeks of discrete estrogen treatment and external observations of hair regrowth adopted in these works make it difficult to point out the exact alteration of hair cycle [26], [27]. In the present work, based on the detection of hair cycle alteration, we further explored the signaling mechanism. Through time-lapse morphology assay, our present work elaborates that estrogen caused premature catagen and subsequently sustained telogen. On molecular level, we found that TGF β2 functioned as an estrogen downstream effector to trigger premature onset of catagen, and then BMP4 increased. The up-regulation of BMP4 may further function to prevent anagen transition and maintain telogen. Moreover, the present study also confirmed that the suppressive effect of estrogen on hair cycle is reversible, owning to retention of HFSCs. The DP signature gene expressions suggested the reservation of the hair inductive potential (Figure 7).

In clinical dermatology, alopecias are divided into scarring and nonscarring categories on the basis of different statuses of HFSCs. Destruction of HFSCs leads to the permanent hair loss named scarring alopecia with no chance of trichogenesis. In contrast, if HFSCs were kept undamaged in their niche, the hairs could regrow and the alopecias could be cured [28]. In the present work, the hair regrowth after estrogen withdrawal, together with the expression of the HFSCs markers after 15 days of estrogen administration demonstrated that although estrogen arrested hair cycle, it did not ablate HFSCs. The signature genes of DP function in triggering anagen. These gene expressions were not compromised upon estrogen. All the observations suggested that the clinical estrogen caused alopecia might be a nonscarring type of alopecia and could be cured.

Catagen is the regressive stage in the lifespan of HF and is an exactly controlled process of coordination between cell differentiation and apoptosis [38]. During spontaneous catagen, apoptosis happens not only to regressive proximal follicle epithelium but also to the central inner root sheath, the bulge, and the secondary germ [13], [39]. Compared with the spontaneous catagen, the premature catagen induced by estrogen is more like a hard braking. That is why the precortex cells as the immediate terminally differentiated cells [40] underwent apoptosis firstly.

DP is an inductive mesodermal structure which sends and receives morphogenetic signals inducing HFSCs to proliferate or to differentiate. Intriguingly, DP functions as not only anagen initiator but also catagen inducer [16]. We speculate that the ER α signaling cross-talks with the normal signaling in DP and up-regulates TGF β2 after estrogen binds and activates ER α residing at DP. Responding to the signal alterations, the precortex cells are committed to programmed cell death.

Previous report showed that estrogen inhibitor ICI 182780 causes advanced hair growth [26], [41]. Combining with our results, we suppose that even as low as physiological concentration of serum estrogen could play a suppressive effect on hair cycle, until the extrinsic ICI 182780 counterbalances the suppressive effect of estrogen and HFs perform advanced hair growth consequently. Thereby, HFs may be sensitive to the estrogen of a concentration much lower than the pregnancy plasma level and the doses in the published murine models. Accordingly, estrogen might be among the basic modulating elements of normal hair cycling.

## Materials and Methods

### Animals

ER α null mice (Stock Number: 004744) were purchased from The Jackson Laboratory (Bar Harbor, ME, USA) and genotyped following the corresponding proposal. Male CD1 mice were purchased from Vital River Laboratories (Beijing, China). The mice were bred as specified-pathogen-free (SPF) and given food and water *ad libitum*. The surgeries and sacrifices were performed under Avertin anesthesia, and every effort was made to minimize suffering. All procedures performed in this study were in accordance with instructions and permissions of the ethical committee of the Institute of Zoology, Chinese Academy of Sciences. The ethical committee had approved this study and the Permit Number is IOZ12001.

### Hair Regrowth Assay

The 17β-estradiol (Sigma, St. Louis, MO, USA) was dissolved in sesame oil (Sigma) at a concentration of 6.6 µg/ml and was s.c. injected as 100 µl/mouse/day. The control group was injected the same volume of sesame oil. Dorsal hairs of all the mice were shaved after 15 days of continuous injection completed (P54). The appearance of hair regrowth was photographed ten days after hair shaving.

In the rescue assay, the specific inhibitor of TGF β signaling, SB431542 (Merck, Darmstadt, Germany) or vehicle (DMSO) was i.p.-injected 1 h before estrogen administration for 4 days.

### Histological Analysis and Immunofluorescence Staining

Frozen longitudinal skin sections were prepared. 40 µm sections were used in time-lapse morphology analysis following routinely stained with haematoxylin and eosin and 10 µm sections were used in immunofluorescence staining. To perform the immunofluorescence staining, frozen sections were fixed in 4% paraformaldehyde. Fixed sections were treated with 0.5% Triton X-100 for 15 min at room temperature and then blocked with 5% bovine serum albumin for 1 h at 37°C. After rinsing with PBS, the sections were incubated in a humidified chamber at 4°C overnight with primary antibody. Sections were rinsed with PBS for 4 times and then incubated in dark for 1 h at 37°C with appropriate fluorescence-labeled secondary antibodies (1∶100, all from Zhongshan Gold Bridge Biotechnology, Beijing, China). The sections were counterstained with propidium iodide (PI) for 10 min at room temperature. After rinsed with PBS for 4 times, the sections were examined and photographed. Primary antibodies used were as follows: FITC conjugated rat-anti-alpha-6 integrin (Biolegend, San Diego, USA), FITC conjugated rat-anti-CD34 (eBioscience, San Diego, USA), rabbit-anti-ER α (Epitomics, Burlingame, USA), rabbit-anti-LGR5 (kindly gifted by Dr Aaron J W Hsueh, Stanford University School of Medicine), rabbit-anti-Ki67 (Zhongshan Gold Bridge Biotechnology), rabbit-anti-BMP4 (Abcam, Cambridge, UK), rabbit-anti-phospho-Smad 1/5 (Cell Signaling Technology, Boston, MA, USA), rabbit-anti-TGF β2 (Santa Cruz, Santa Cruz, CA, USA), rabbit-anti-cleaved caspase 3 (Cell Signaling Technology, Boston, MA, USA). TUNEL assay was performed using tunnel assay kit (Roche, Indianapolis, IN, USA) according to the manufacturer’s instructions.

### Cell

CD133 positive DP cells were obtained from neonatal mice by cell sorting, following the protocol previously reported [34]. Briefly, the skins were derived from neonatal mice. Dermis was separated from epidermis using trypsin and dispase. The dermis was digested into single cells and processed by fluorescence activated cell sorting (FACS) to obtain CD133 positive cells (FITC labeled-CD133 antibody, eBioscience, San Diego, CA, USA). The primary cells were cultured in AmnioMax-C100 media (Invitrogen, Carlsbad, CA, USA). Taken the estrogen mimic effect of phenol red into consideration, when the cells were passaged and exposed to estrogen treatment, they were transferred to phenol red free DMEM/F12 1:1 medium (Invitrogen) with bFGF (PeproTech, Rocky Hill, NJ, USA). After the first passage, the cells were seeded on 6-well plates. The cells adhered to the plates one day after and were treated with 17β-estradiol (Sigma) or 17β-estradiol together with ER down-regulator ICI 182780 (Sigma) for 24 hours and were harvested for RNA extraction.

### Immunocytochemistry

The DP cells on cover glasses were fixed in 4% paraformaldehyde and penetrated with 0.1% Triton X-100. After blocking with 5% bovine serum albumin, cells were incubated in a humidified chamber at 4°C overnight with primary antibody. After incubated with the appropriate FITC-labeled secondary antibodies (1∶100), the cells were counterstained with PI. Primary antibodies used in immunocytochemistry were as follows: rabbit-anti-SMA α (Abcam), rabbit-anti-Collagen I (Abcam) and rabbit-anti-ER α (Epitomics).

### Quantitative PCR

DNA-free RNA was extracted from the differently treated DP cells using RNeasy Micro Kit (Qiagen, Valencia, CA, USA). Reverse transcription was performed with SuperScript III Reverse Transcriptase kit (Invitrogen). Real-time PCR was performed using 2× SYBR Green quantitative PCR master mix (Promega, Madison, WI, USA), 1 µl template cDNA, and 1 µl primers. GAPDH was used as normalization. Real-time PCR was processed on Roche LightCycler 480. Primer sequences for real-time PCR were from previous report [32] or primer bank (http://pga.mgh.harvard.edu/primerbank/).

### Statistical Analysis

All data was presented as mean ± SEM. Results were analyzed by independent sample T test or one-way ANOVA.

## Supporting Information

Figure S1 **HFs of the estrogen treated mice showed activation of BMP pathway (lower amplification, the fourth day of treatment).** (A) BMP4 was detected in dermal papillae of the estrogen treated WT mice, while it’s almost undetectable in the vehicle oil treated WT and the estrogen treated ER α (−/−) mice. (B) BMP activity was further demonstrated by P-Smad 1/5 immunofluorescence. The estrogen treated WT mice had significant staining in precortex cells, while the vehicle oil treated ones and the estrogen treated ER α (−/−) had negligible staining in ORS. The asterisk indicates non-specific staining of hair shaft or lipid. DP: dermal papilla; PC: precortex; E2: 17β-estradiol; HF: hair follicle; ER: estrogen receptor. Scale bar: 50 µm.
(TIF)
