# Supplementary figures and images for: Estrogen Leads to Reversible Hair Cycle Retardation through Inducing Premature Catagen and Maintaining Telogen
Source: PLoS One. 2012 Jul 5;7(7):e40124. doi: 10.1371/journal.pone.0040124 (PMC3390338; doi:10.1371/journal.pone.0040124)

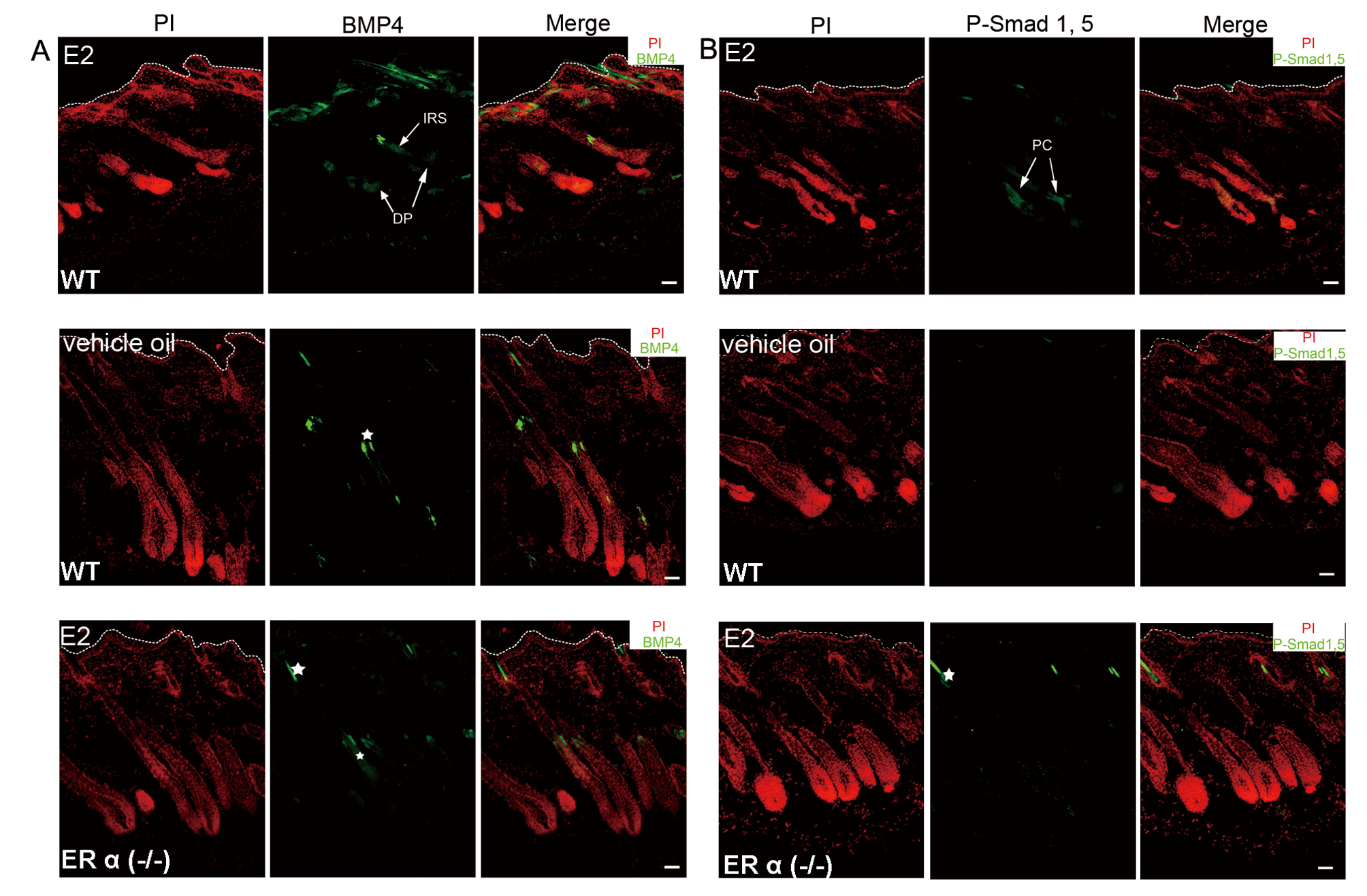

Supplement: Figure S1 — HFs of the estrogen treated mice showed activation of BMP pathway (lower amplification, the fourth day of treatment). (A) BMP4 was detected in dermal papillae of the estrogen treated WT mice, while it’s almost undetectable in the vehicle oil treated WT and the estrogen treated ER α (−/−) mice. (B) BMP activity was further demonstrated by P-Smad 1/5 immunofluorescence. The estrogen treated WT mice had significant staining in precortex cells, while the vehicle oil treated ones and the estrogen treated ER α (−/−) had negligible staining in ORS. The asterisk indicates non-specific staining of hair shaft or lipid. DP: dermal papilla; PC: precortex; E2: 17β-estradiol; HF: hair follicle; ER: estrogen receptor. Scale bar: 50 µm. (TIF) [file pone.0040124.s001.tif]
